# Toward Sensor-to-Text Generation: Leveraging LLM-Based Video Annotations for Stroke Therapy Monitoring

**DOI:** 10.3390/bioengineering12090922

**Published:** 2025-08-27

**Authors:** Mohammad Akidul Hoque, Shamim Ehsan, Anuradha Choudhury, Peter Lum, Monika Akbar, Shashwati Geed, M. Shahriar Hossain

**Affiliations:** 1 Department of Computer Science, The University of Texas at El Paso, El Paso, TX 79968, USA; sehsan@miners.utep.edu (S.E.); achoudhury@miners.utep.edu (A.C.); mhossain@utep.edu (M.S.H.); 2 Department of Biomedical Engineering, The Catholic University of America, Washington, DC 20064, USA; 3 Department of Physical Therapy and Movement Sciences, The University of Texas at El Paso, El Paso, TX 79968, USA; sgeed@utep.edu

**Keywords:** large language model, machine learning, stroke therapy, deep learning

## Abstract

Stroke-related impairment remains a leading cause of long-term disability, limiting individuals’ ability to perform daily activities. While wearable sensors offer scalable monitoring solutions during rehabilitation, they struggle to distinguish functional from non-functional movements, and manual annotation of sensor data is labor-intensive and prone to inconsistency. In this paper, we propose a novel framework that uses large language models (LLMs) to generate activity descriptions from video frames of therapy sessions. These descriptions are aligned with concurrently recorded accelerometer signals to create labeled training data. Through exploratory analysis, we demonstrate that accelerometer signals exhibit distinct temporal and statistical patterns corresponding to specific activities, supporting the feasibility of generating natural language narratives directly from sensor data. Our findings lay the foundation for future development of sensor-to-text models that can enable automated, non-intrusive, and scalable stroke rehabilitation monitoring without the need for manual or video-based annotation.

## 1. Introduction

Worldwide, stroke is a leading cause of long-term disability [1,2]. A majority of patients survive the initial neurological event, but experience persistent upper and lower-extremity motor impairment, weakness, coordination deficits, and muscle spasticity that limits their independence in everyday activities and instrumental activities of daily living (ADL/IADLs) [3,4,5].

Post-stroke physical and occupational therapy focuses on neurorehabilitation to help patients recover lost motor function and regain independence in ADL/IADLs. A crucial component of the therapeutic dose in neurorehabilitation is the amount of time the impaired limb spends in meaningful task-specific practice [6,7,8,9]. Yet, therapists lack clear measures to quantify the duration and intensities of this dosing component. Currently, the dose of therapy is quantified as the total duration of a therapy session, with large variations in the actual amounts of time spent on the active ingredients of therapy: *time spent in meaningful task-specific training*, and the *intensity/progression* of this training.

Recent years have seen the emergence of wearable sensors and video recordings, coupled with machine learning (ML) and artificial intelligence (AI) tools to better understand the real-world use of the impaired upper extremity [10,11]. A clinically significant measure of motor recovery after a neurological injury is how much the patient actually uses their impaired limb in their own ecosystem for activities that are meaningful to them [12]. These emerging technologies offer the potential to monitor movement in real-world settings, allowing therapists to tailor treatment plans based on personalized, data-driven insights from real-world arm use. However, significant challenges remain in integrating multimodal sensor data, ensuring data quality and consistency, and developing robust models that can generalize across patients and environments.

Accelerometers are lightweight, non-intrusive devices used to monitor limb movements [13,14,15,16,17], gait [18,19,20], and balance [19,21,22] in neurorehabilitation. Wrist-worn accelerometers provide real-time data on upper extremity movements by measuring acceleration along multiple axes. Conventional wrist-worn accelerometry has been used to quantify upper extremity movement in stroke [13,23,24,25], cerebral palsy [16,26,27], spinal cord injuries [28], and radial wrist fracture recoveries [14]. However, it remains challenging to distinguish between specific movement types (e.g., lifting, grasping) solely on the basis of accelerometer data. Accelerometry-based activity recognition relies on manual annotation of concurrently recorded video frames for ground truth. This frame-by-frame manual annotation of video data is time-consuming, which limits widespread clinical adoption of current wrist-worn accelerometry approaches despite their clinical validation.

This paper presents a video-driven pipeline to automatically annotate accelerometry data to minimize the need for human annotation. We leverage Large Language Models (LLMs) to generate descriptive text from video frames, which is then used to train machine learning models for identifying specific tasks (e.g., folding laundry, typing, walking) performed by participants. Additionally, we conduct an exploratory analysis of wrist-worn accelerometer data in healthy controls and patients with stroke, highlighting distinctive features and patterns that can support automated detection of various ADLs/IADLs that form the basis of task-specific training and clinical assessments. The contributions of this paper are as follows:

- We propose an automated video-based annotation pipeline that integrates LLMs and ML techniques to detect and classify patient activities during therapy sessions.
- We explore the feasibility of identifying task-specific distinctive features from accelerometer signals.
- We propose a deep learning-based visionary framework that can deliver text-based feedback to patients and clinicians using multimodal data, enhancing stroke rehabilitation monitoring.

The rest of the paper is organized as follows: Section 2, Section 3 and Section 4 discuss the relevant literature, the description of the problem, and the proposed methodology, respectively. Section 5 outlines the experimental results. In Section 6, we provide a general discussion of the proposed model and conclude the paper in Section 8.

## 2. Literature Review

Rehabilitation has been through a significant evolution from laboriously manual and human subjective practices to welcoming wearable devices and different technologies during previous decade. This transformation was made possible not only by researchers but also through the significant contributions of clinicians, who were committed to bringing these advancements into real-life settings. The initiative to automate activity annotation from patient video data in stroke therapy, as highlighted in this study, is vigorously grounded in this wider movement toward real-world, technology-driven rehabilitation. Clinicians and researchers have always been investigating cutting-edge ideas and techniques to automate the manual work part of this process.

**Video description generation:** Early approaches of video description generation were dependent on classical template-based methods, where subjects, verbs, and objects were detected separately and assembled into sentences using fixed grammar templates. Khan et al. proposed a star skeleton model and trained Hidden Markov Models (HMM) to classify actions like standing, walking, and running [29]. They used Haar features for object detection, edge and color histograms for scene classification, and a template-based method to generate natural language descriptions from extracted features. Hanckmann et al. proposed a hybrid system combining a bag-of-features action classifier with a rule-based description generator to create sentences for 48 human actions [30]. The shift to deep learning for video description began around 2015–2016 with encoder-decoder models using Convolutional Neural Networks (CNNs) for feature extraction and Recurrent Neural Networks (RNNs), including Long Short-Term Memory networks (LSTMs) for sentence generation. Here, CNNs process the visual content to learn spatial patterns, while RNNs and LSTMs interpret the sequence of extracted features to generate coherent textual descriptions over time. Ramanishka et al. proposed the MMVD model to generate rich video descriptions [31]. Hori and their team used a sequence-to-sequence encoder-decoder model with LSTM networks, enhanced by a temporal attention mechanism and a newly proposed multimodal attention fusion model [32]. Thomason et al. proposed a Factor Graph Model that integrates visual detection scores and language statistics to select the best subject-verb-object-place (SVOP) combination for video description [33]. Also, Wang et al. proposed a Hierarchical Reinforcement Learning (HRL) framework where a high-level Manager LSTM sets goals and a low-level Worker LSTM generates words to create fine-grained video captions [34]. A deep semantic framework combining scene-adaptive key frame extraction, local event description, and video-text summarization through reinforcement learning was investigated by [35]. Chen et al. proposed ShareGPT4Video, using a Differential Sliding-Window Captioning (DiffSW) strategy with GPT-4V to generate detailed and temporally accurate video captions [36].

**Activity detection from video:** The evolution of activity recognition from video started with handcrafted feature methods in the early 2000s. More recently, Allappa et al. proposed using SVMs with new sequence kernels to classify activities from videos represented as sequences of feature vectors [37]. A multi-stream deep CNN approach was proposed by De et al. that processes three types of input (i.e., RGB frames, optical flow images, and visual rhythm images) [38]. Kulbacki et al. proposed a real-time framework for human activity detection and recognition in outdoor video surveillance, using an improved tracking method based on frame differencing, feature correlation, and a robust hypothesis-verification approach [39]. Two-Stream CNNs also showed strong performance, especially for complex motion patterns [40]. A hybrid deep learning model is proposed by SOleimani et al., activity recognition framework using a fine-tuned GPT-2 model, and a custom Transformer architecture [41].

**Activity detection using accelerometers:** Lara et al. surveyed human activity recognition (HAR) using wearable sensors [42]. HAR applications span fitness, healthcare, rehabilitation, gait analysis, and assistive technologies. ML and Deep Learning (DL) models are compared by Shakya et al. for HAR using smartphone accelerometer data, finding that while classical ML methods like KNN achieved high accuracy, CNNs performed better [43]. They also showed that using multiple body sensors improves recognition performance over a single sensor. Chernbumroong et al. investigated using a single wrist-worn accelerometer to classify five daily activities: sitting, standing, lying, walking, and running [44]. They compared Decision Tree C4.5 and Artificial Neural Network classifiers across four feature sets and found that the decision tree consistently outperformed the neural network. Panwar et al. proposed a CNN-based model for recognizing three fundamental arm movements using data from a single wrist-worn accelerometer [45]. Their method eliminated the need for manual feature extraction by using deep learning and achieved a decent accuracy under cross-validation, outperforming conventional methods like SVM, LDA, and K-means clustering.

Overall, advancements in video description, video-based activity detection, and accelerometer-based recognition have transformed human activity monitoring from manual, labor-intensive methods to intelligent, automated systems.

## 3. Problem Description

A typical post-stroke upper extremity rehabilitation session involves task-specific practice of everyday activities such as meal preparation, dressing, feeding, grooming, managing medications with the goal of improving independence in ADL/IADLs while addressing the individual’s specific sensorimotor impairments. For this study, two data streams are collected in parallel—(a) accelerometer data, and (b) video data. The accelerometer data is represented as: $T=\{a_{1},a_{2},\ldots ,a_{n}\}$, where $a_{i}=\left(x_{i},y_{i},z_{i}\right)$ represents the accelerometer reading at time *i*. The accompanying video stream is represented as $V=\{v_{1},v_{2},\ldots ,v_{m}\},$ where two consecutive frames $v_{j}$ and $v_{j+1}$ are recorded at certain intervals (e.g., a second).

Let $f_{\theta }$ be the learned mapping function from accelerometer data to activity descriptions:

$$f_{\theta }:T=\left\{a_{1},a_{2},\ldots ,a_{n}\right\}\to S=\left\{s_{1},s_{2},\ldots ,s_{k}\right\},$$

where $s_{j}$ denotes a label, text, or summary of the generated activity narrative.

To learn $f_{\theta }$, we leverage the video sequence *V* by creating a function *g* that maps *V* to a textual sequence:

g:V→S,

providing activity descriptions. These descriptions are aligned with the corresponding accelerometer sequence *T*, producing training pairs (T,S) for learning $f_{\theta }$.

The purpose of this paper is to validate some of the mapping aspects of this problem.

## 4. Methodology

**Figure 1:**
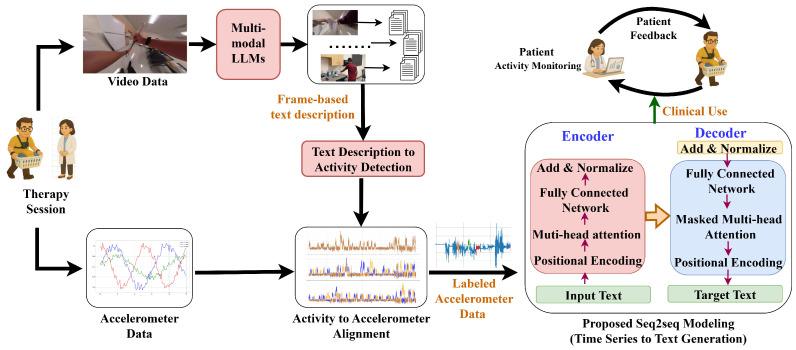
The proposed methodology consists of several modules: video and accelerometer data are collected from therapy sessions, video is processed using LLMs and ML techniques to generate frame-based activity descriptions and labels. These activity labels are then aligned with the accelerometer data, which can be used to train a Seq2Seq model for time-series-to-text generation, supporting clinical feedback and patient monitoring.

In this paper, we propose a workflow to learn a generative mapping function $f_{\theta }$ that produces narrative descriptions of activities from accelerometer data. The training data, consisting of annotated text aligned with accelerometer snippets, is constructed using video and accelerometer streams collected in parallel during a study session. Figure 1 illustrates the overall workflow.

The workflow pipeline begins with the collection of video and accelerometer data from neuro-rehabilitation therapy sessions involving participants diagnosed with a history of stroke, confirmed via neuroimaging. As illustrated in Figure 1, a multimodal LLM can be employed to generate textual descriptions and those descriptions can then be analyzed to detect activity classes. Further details on this process are provided in Section 4.2.

The LLM-generated (or any machine learning-based) textual labels from the video can be temporally aligned with the wrist-worn accelerometer data, as both modalities are recorded concurrently. This alignment enables the creation of a labeled accelerometer dataset, where each time segment is annotated with a corresponding activity description.

As shown in Figure 1, we propose a sequence-to-sequence (Seq2Seq) transformer model to learn a generative function that maps accelerometer signals to textual activity narratives. Details of the proposed model architecture are discussed in Section 4.3. The following subsections describe the components of the proposed workflow.

### 4.1. Data Sources

The dataset utilized in this study comprises two distinct cohorts: (1) individuals with neuroimaging-confirmed stroke diagnoses, and (2) healthy controls without any known neurological impairments. Patients with stroke were recruited from the community using recruitment flyers. The inclusion criteria were, (1) neuroimaging-confirmed stroke, (2) age more than 18-years old at the time of informed consent, (3) no known orthopedic or neuromuscular injuries that limited completion of study tasks, (4) Mini-Mental status exam score >24. Individuals were excluded if they (1) exhibited neglect (asymmetry >3 errors on the Mesulam’s symbol cancellation test), (2) prior stroke with residual motor impairment, (3) received botulinum toxin during study participation. Similar criteria applied for healthy controls with the exception of a stroke diagnosis. The study was conducted in accordance with the Declaration of Helsinki and approved by the Institutional Review Board of The University of Texas at El Paso (protocol number: 2127649, approval date: 4 February 2024). Written informed consent was obtained from all individuals tested in the study.

Healthy control data primarily served technical validation purposes, such as evaluating the feasibility of recognizing activity patterns and generating textual descriptions from video frames using multimodal LLMs. In contrast, the data from stroke patients were used to analyze temporal activity patterns in relation to clinical impairment. The patient dataset enabled the investigation of how extracted features from accelerometer data correlate with functional activities, offering insights into the potential of wearable sensing for neuro-rehabilitation.

All study participants completed a structured activity script [13,23] comprised of activities and instrumental activities of daily living in a simulated apartment (Activities of Daily Living Lab, Department of Occupational Therapy at UTEP). This simulated apartment includes a functional kitchen, bedroom, laundry area, storefront, and dining area. Specifically, participants completed a (1) laundry, (2) linen management, (3) grocery shopping, (4) meal preparation, (5) financial management, 180 (6) medication management, and (7) typing task.

Throughout these activity script sessions, two data streams were collected simultaneously: video 182 recordings and accelerometer data. Video was captured using a chest-mounted camera Kodak PixPro SP360 4K camera (JK Imaging Ltd., Gardena, CA, USA) operating in fish-eye mode to maximize the field of view. Simultaneously, hand movement data were collected using wrist-worn accelerometers (ActiGraph CentrePoint Insight Watch) on both wrists. To enable the offline synchronization of video and accelerometry data streams, the experimenter placed both watches at rest on a stable surface for 2 min, followed by moving the accelerometers up and down (vertically) 5 times, followed by a second 2-min rest on stable surface in view of the video before placing the watches on the patients’ hands at the start of the activity script. A similar sequence of rest-vertical movement-rest was followed after removing the watches from the patients’ hands at the end of data collection. This rest-movement-rest sequence in accelerometry data created a stereotypical pattern easily identified for offline synchronization between video and accelerometry data.

The video footage was annotated frame by frame by trained research assistants using the FAABOS (Functional Arm Activity Behavioral Observation System) coding scheme [46]. Each video frame was labeled according to the five FAABOS categories, which were subsequently collapsed into three categories (functional, non-functional, or unknown hand use). Functional activities included any reaching to grasp or prehension, such as pushing open a door, opening or closing jars, etc. (purposeful upper-extremity activities contributing to the desired task). Non-functional periods included rest and arm movements associated with gait, gestures, and purposeless movements not directly related to the desired task. Although these “non-functional” tasks reflect meaningful activity, they were considered non-functional in this context as they were not prehension, which is the target of interventions in conventional upper-extremity stroke rehabilitation trials. The right and left upper extremities were coded separately by two annotators independently. Any discrepancies were resolved by a third trained annotator (majority vote). In addition, each video frame was also labeled with the corresponding activity (e.g., laundry, meal preparation), with the upper right and left limbs coded independently. Participants completed the activities naturally, without specific instructions regarding arm usage or task execution strategy, and were encouraged to behave as they would at home. The duration of the sessions ranged from 30 to 60 min.

### 4.2. Text Annotation from Video

Automatic annotation of video data in the context of neuro-rehabilitation involves two major components: (1) classifying video frames into task-relevant categories, and (2) generating textual descriptions using a multimodal LLM. The classification component itself can be approached in two distinct ways: one involves direct classification based on visual features extracted from individual frames, and the other leverages image-to-text generation via multimodal LLMs, followed by task classification using the generated text. Given the challenges associated with accurate task classification from raw visual features, especially in complex, cluttered scenes. The latter approach of generating textual descriptions and subsequently using them for classification proves to be more robust and semantically informative.

While task classification offers a direct path for temporally labeling the video data and aligning it with the corresponding segments of accelerometer data, an alternative and more compelling research direction involves describing video content in natural language over time and transferring these descriptions to the accelerometer domain. From an application standpoint, learning to generate text descriptions directly from accelerometer signals at inference time offers significant potential, not only for neuro-rehabilitation monitoring but also for broader use cases in assistive technology, occupational health, and behavior recognition.

Recent advances in LLMs have shown significant promise in recognizing and describing human activities from visual data. By leveraging their multimodal reasoning capabilities, these models can generate frame-level descriptions of patient activities with remarkable contextual understanding. However, processing every individual video frame through an LLM is computationally intensive and becomes impractical for extended recordings. To address this challenge, videos are first decomposed into individual frames, and then a single representative frame, sampled at regular intervals, is selected. This strategy provides a computationally efficient approximation while preserving sufficient temporal granularity to describe the activities meaningfully.

There are several open-source large language models (LLMs) available for image-to-text conversion, as summarized in Table 1. Notable examples include BLIP-2 and LLaMA-3. BLIP-2 (Bootstrapping Language-Image Pre-training) is specifically designed to bridge vision and language modalities, making it particularly effective at generating detailed and contextually rich captions from visual inputs. Although LLaMA-3 is primarily a text-based language model, it can be integrated into multimodal frameworks with vision encoders to enable high-quality image-to-text generation.

In addition to open-source models, several cloud-based APIs are available for multimodal inference, including those provided by OpenAI, Anthropic, and Google Gemini. OpenAI’s models such as GPT-4o are widely recognized for their ability to produce high-quality, context-aware textual outputs, making them suitable for automatic annotation tasks. However, due to the sensitive nature of patient data, the use of commercial platforms may be restricted in research contexts where privacy and data confidentiality must be strictly maintained. Section 5 describes some experiments relevant to multimodal LLMs for text generation.

### 4.3. Seq2seq Learning Model

To generate text from accelerometer time series data, the proposed framework requires a specialized transformer-based architecture, specifically a decoder-style model capable of autoregressive text generation conditioned on input time series segments. Off-the-shelf foundation models, such as GPT or LLaMA, are not directly applicable in this setting, as they are not pre-trained to handle continuous multivariate time series as input. Consequently, the framework must either (1) train a transformer model from scratch using appropriate time series embeddings, or (2) adapt an existing language model through techniques such as Low-Rank Adaptation (LoRA) [54], combined with a time series encoder module that transforms sensor data into a format suitable for textual generation.

Recent efforts like Informer [55] have explored transformer-based architectures for time series forecasting, but they are not designed for text generation, rather for forecasting time series. Therefore, to support narrative generation from accelerometer signals, our framework necessitates a tailored sequence-to-sequence transformer design, either trained end-to-end or adapted from pretrained general-purpose language models with time series-specific encoders.

Another important consideration is that the length of time series segments directly impacts both the granularity of generated text descriptions and the resulting analytical insights. To address this, we propose a variable-length windowing strategy that constructs a hierarchical structure over the time series. This hierarchy will support multiple levels of abstraction ranging from fine-grained descriptions of short segments to higher-level summaries of longer intervals. At the top of the hierarchy, the root node will provide a comprehensive summary of the entire accelerometer time series for a given session.

## 5. Experimental Results

The experiments in this section primarily assess the feasibility of preparing training data involving video, accelerometer signals, and textual descriptions, as well as validating whether accelerometer data contain informative patterns relevant to activity recognition. In addition, we evaluate the effectiveness of some of the existing multimodal tools in generating meaningful text from video frames. These preliminary results help establish the technical viability of the core components of the framework, feature extraction and text generation.

The questions we seek to answer through the experiments are as follows.

- How effectively can relevant activity descriptions be generated from videos using locally deployed LLMs? (Section 5.1)
- What are the strengths and limitations of cloud-based LLMs in generating structured activity descriptions from video inputs? (Section 5.2)
- To what extent are certain human activities detectable using text descriptions of video frames generated by LLMs? (Section 5.3)
- To what extent accelerometer data can be used for identifying different human activities? (Section 5.4 and Section 5.5)

### 5.1. Generating Activity Descriptions Using Locally Deployed LLMs

**Figure 2:**
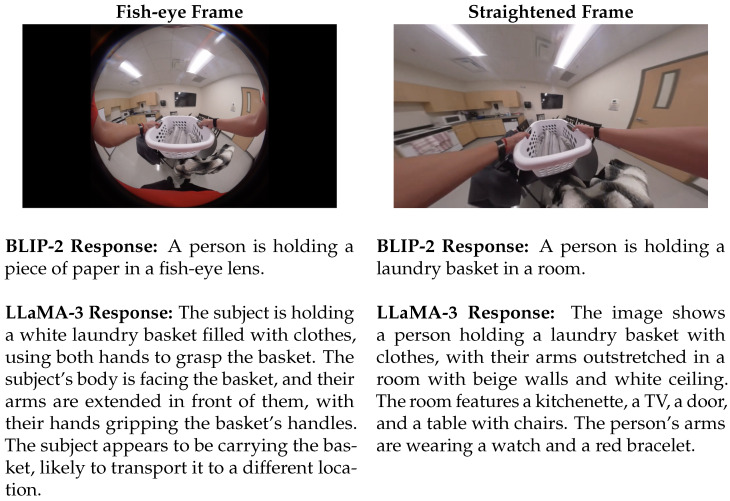
BLIP-2 and LLaMA-3 Responses for (**Left**) a fish-eye frame captured from a chest-mounted camera, and (**Right**) for the straightened frame.

In this section, we investigate the feasibility of generating human-readable textual annotations from individual video frames. The overarching goal is to later transfer these annotations to corresponding segments of accelerometer time series, enabling the training of models that can generate similar descriptions solely from sensor data. The dataset includes video frames captured using either a chest-mounted wide-angle (fisheye) camera or a standard lens camera. Videos recorded with the wide-angle lens exhibited noticeable distortion, which was corrected through a video straightening process prior to analysis (see Figure 2).

The straightened image provides more informative visual features to a multimodal LLM compared to the original fish-eye view, thereby enabling more accurate and contextually relevant text generation. In our preliminary investigation, we evaluated the performance of two models: BLIP-2 and LLaMA-3, on both fish-eye and straightened images. BLIP-2 does not require prompting, as it is inherently designed to generate descriptions directly from visual inputs. In contrast, LLaMA-3 operates as a language model and requires a textual prompt for each input frame. For this experiment, a simple instruction was provided to LLaMA-3, asking it to describe the content of the input image.

As shown in Figure 2, BLIP-2 misclassified the object in the fish-eye frame as a piece of paper, whereas its response to the straightened image correctly identified a laundry basket. Similarly, the response from LLaMA-3 for the straightened image is notably more detailed and semantically rich. It accurately recognized not only the laundry basket and clothing, but also surrounding elements such as the television, room furnishings, and even the wrist-worn accelerometer devices (interpreted as a watch). Compared to BLIP-2, LLaMA-3 provides a more comprehensive and nuanced description when operating on the straightened image.

To guide the model’s output, a prompt was crafted to elicit structured responses in three narrative chunks: hand activity, background elements related to the hands, and a high-level description of the subject’s overall activity. The prompt used is shown below:


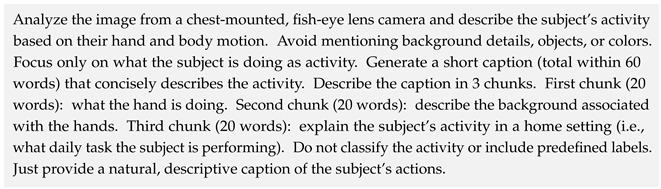


A few examples of descriptions generated by LLaMA-3 for different video frames are presented in Table 2, with the three narrative chunks displayed in separate columns for each image. Each row corresponds to a frame representing a distinct activity – linen management, laundry, self-feeding, financial management (e.g., writing a check), and kitchen-related tasks, respectively. LLaMA-3 successfully responded to the structured prompt, generating coherent and contextually relevant descriptions across all three narrative components.

For instance, the first row in Table 2 shows a frame associated with linen management, where the patient is folding clothes. The “hand activity” chunk accurately describes the motion as “grasping a piece of fabric”, a meaningful indicator of functional recovery, particularly important for therapists monitoring hand use post-stroke. The “background” chunk correctly identified the surroundings as a bedroom with a bed and dresser. While the final “activity description” chunk refers to the task as changing clothes, which is slightly inaccurate, it still remains contextually close to the linen management theme. Such minor discrepancies could be mitigated by using additional frames at finer temporal intervals and allowing the LLM to summarize over multiple frames.

Overall, the remaining examples are also reasonably well described by LLaMA-3, demonstrating its potential as a local multimodal tool for automatic video annotation. This approach could significantly reduce the extensive manual effort typically required by research assistants for frame-by-frame annotation.

### 5.2. Generating Activity Descriptions Using Cloud-Based LLMs

In addition to evaluating locally deployed LLMs, we assessed how well GPT-4o, a cloud-based LLM developed by OpenAI, generates activity descriptions. Due to privacy concerns related to the transmission of patient data over the Internet and the risk of potential storage on third-party servers, we excluded patient recordings from this evaluation. With appropriate contractual agreements in place to ensure privacy protection, the use of cloud-based LLMs becomes a feasible option for commercialization of deployed applications. For our experiments, we conducted separate sessions with three healthy volunteers, instead of patients, in the same controlled laboratory environment. Exemplary data from one healthy control are described in this section. Frames were used as input to GPT-4o along with prompts to examine the feasibility of employing cloud-based LLMs for activity annotation.

The prompt used to query a multimodal LLM can significantly influence the quality and structure of the generated response. Prompts may range from generic instructions such as “Describe the frame” to precise queries that generate structured outputs. In this study, we used two types of prompts: the first was a general open-ended instruction, while the second was more targeted, asking the model to identify which hand was grasping which object. The prompts are as follows.


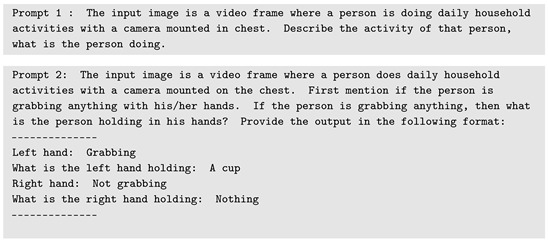


Table 3 presents the outcomes of the two prompts for three different frames relevant to three activities: self-feeding, laundry, and picking up a bottle. For the first image in Table 3, Prompt 1 generates a general yet accurate description of the activity, correctly identifying that the person is opening a plastic container. In contrast, Prompt 2 produces a more structured response, specifying the action in terms of which hand is grasping, indicating that a lid and a container are being held. This level of detail is particularly significant, as functional grasping and holding are important indicators of motor recovery, as discussed in Section 5.1.

For the second frame, Prompt 1 also produces a generally accurate response, identifying that the person is doing laundry and carrying a basket filled with clothes. Prompt 2 provides a more detailed interpretation, correctly noting that the container of clothes is held with both the left and right hands.

In the third frame, where the volunteer is holding a water bottle, Prompt 1 provides a general description that includes some contextual details about the kitchen setting. In contrast, Prompt 2 explicitly identifies that the left hand is holding a bottle while the right hand is not engaged in grasping. This level of specificity, indicating which hand is actively involved in the task, can serve as a valuable cue for therapists in assessing functional use of the limbs and determining the appropriate dosage of therapy or rehabilitation exercises.

The findings in this section highlight the potential of large models like GPT-4o to generate highly detailed activity descriptions, demonstrating their suitability for commercial applications. However, privacy concerns must be carefully addressed as organizations move toward deploying such cloud-based models.

### 5.3. Automating Task Annotations

Text generation using large language models (LLMs) presents a promising strategy for classifying images based on task-relevant content, an essential step in analyzing patient activities in rehabilitation settings. The training data required for this approach can be derived from multiple sources, including: (1) manually annotated video frames labeled by research assistants, serving as ground truth, and (2) labels inferred from textual descriptions generated by LLMs. In this section, we report experimental results based on research assistant–annotated frames. For each annotated frame, a textual description was generated using LLaMA-3, our locally deployed LLM, prompted with a general instruction to describe the image. These class labels can subsequently be transferred to the corresponding segments of accelerometer data, enabling the labeling of time series for downstream modeling.

**Figure 3:**
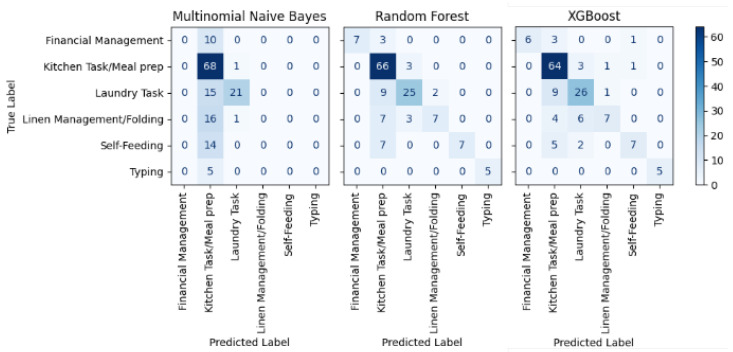
Confusion matrices showing the classification performance of Multinomial Naive Bayes, Random Forest, and XGBoost across six activity categories.

The labels for the frames used in this section are: (1) laundry, (2) linen management, (3) kitchen task, (4) self-feeding, (5) financial management, (6) typing. The number of frames in the video for which manual annotations were present was 754. We used 5-fold cross-validation for three classification techniques: Naive Bayes, Random Forest, and XGBoost. Among the three models, Random Forest shows relatively better performance compared to the other two models, classifying the majority of activities based on LLaMA-3-generated text. The confusion matrix for these models is in Figure 3.

In the confusion matrix, each row represents the true activity labels, while each column corresponds to the predicted labels. For example, in the Random Forest model, the value in the first cell (row 1, column 1) indicates that the model correctly predicted 7 instances as financial management. The adjacent cell (row 1, column 2), with a value of 3, shows that 3 frames actually belonging to the financial management category were misclassified as kitchen tasks.

As shown in Figure 3, the Naive Bayes model achieved the highest number of true positives for the kitchen task. However, it also frequently misclassified other activities—such as laundry, linen management, and self-feeding—as kitchen tasks. Additionally, Naive Bayes struggled to correctly identify activities with lower sample counts, such as linen management, self-feeding, and typing.

In contrast, both the Random Forest and XGBoost models demonstrated stronger performance on underrepresented classes. These models successfully classified the majority of instances for low-frequency activities such as typing and self-feeding, indicating improved robustness in handling imbalanced datasets.

**Figure 4:**
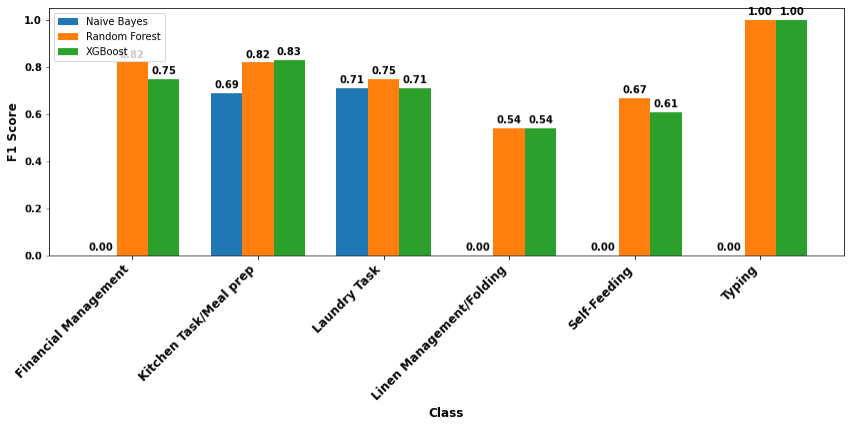
F1 scores of Naive Bayes, Random Forest, and XGBoost classifiers across six activity categories. Random Forest and XGBoost outperform Naive Bayes in all categories, achieving perfect scores for *Typing* and strong performance for *Kitchen Task/Meal Prep* and *Laundry Task*, while Naive Bayes fails on several classes, highlighting its limitations for fine-grained activity classification.

To provide a more comprehensive comparison, the performance of the three models is further evaluated using F1 scores. The F1 score summarizes how well a model balances correct and incorrect results by combining precision and recall into a single metric. A higher F1 score indicates a more reliable and accurate classification, particularly when dealing with imbalanced or unevenly distributed activities. While the confusion matrices suggest that all of the models capture key activity classes to some extent, Random Forest demonstrates a slightly better F1 score. The comparative F1 scores for all models are presented in Figure 4.

The results shown in this section are promising, suggesting that the manual annotation efforts of the research assistants can be scaled by automating parts of the video frame labeling process.

### 5.4. Annotating Accelerometer Data and Finding Temporal Features

**Figure 5:**
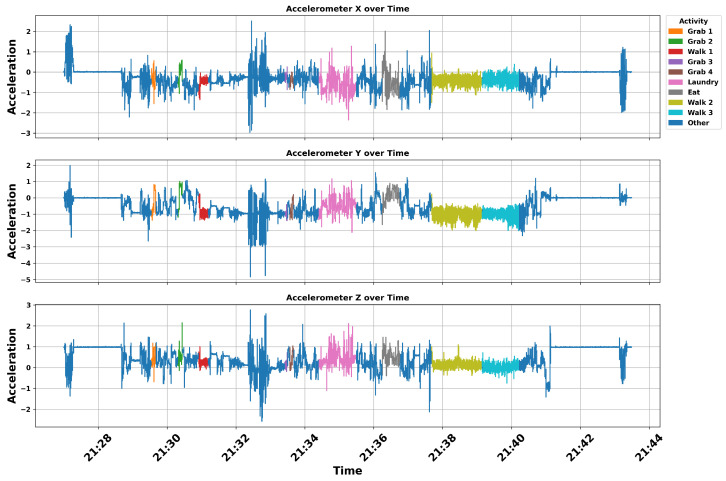
Accelerometer signals along the X, Y, and Z axes over time for a healthy control participant, annotated with activity labels derived from synchronized video data. Distinct colored segments represent functional activities such as doing laundry, eating, as well as non-functional movements labeled as “Other”. This visualization highlights the temporal distribution in acceleration patterns associated with different activities.

To discover features in the accelerometer corresponding to different activities, we first aligned the accelerometer data with the automatically generated activity labels obtained from the video data. Each segment of the accelerometer reading *T* is associated with an activity label $s_{j}\in S$, which creates the mapping $f_{\theta }$. $s_{j}$ can be either a label or text depending on the downstream application. For simplicity, in this section, we use labels as annotations. Figure 5 illustrates the accelerometer signals along the X, Y, and Z axes for a sample activity session annotated by class labels (e.g., first instance of grasping in orange, second instance of grasping in green).

Since it is challenging to visually discern patterns that differentiate activities from raw accelerometer data (e.g., Figure 5), we further analyze the distribution of signal amplitudes observed during scripted tasks. The goal of this analysis is to determine whether the accelerometer data contains distinctive features that can differentiate between task types, or if it primarily captures shared patterns across repeated instances of the same task.

**Figure 6:**
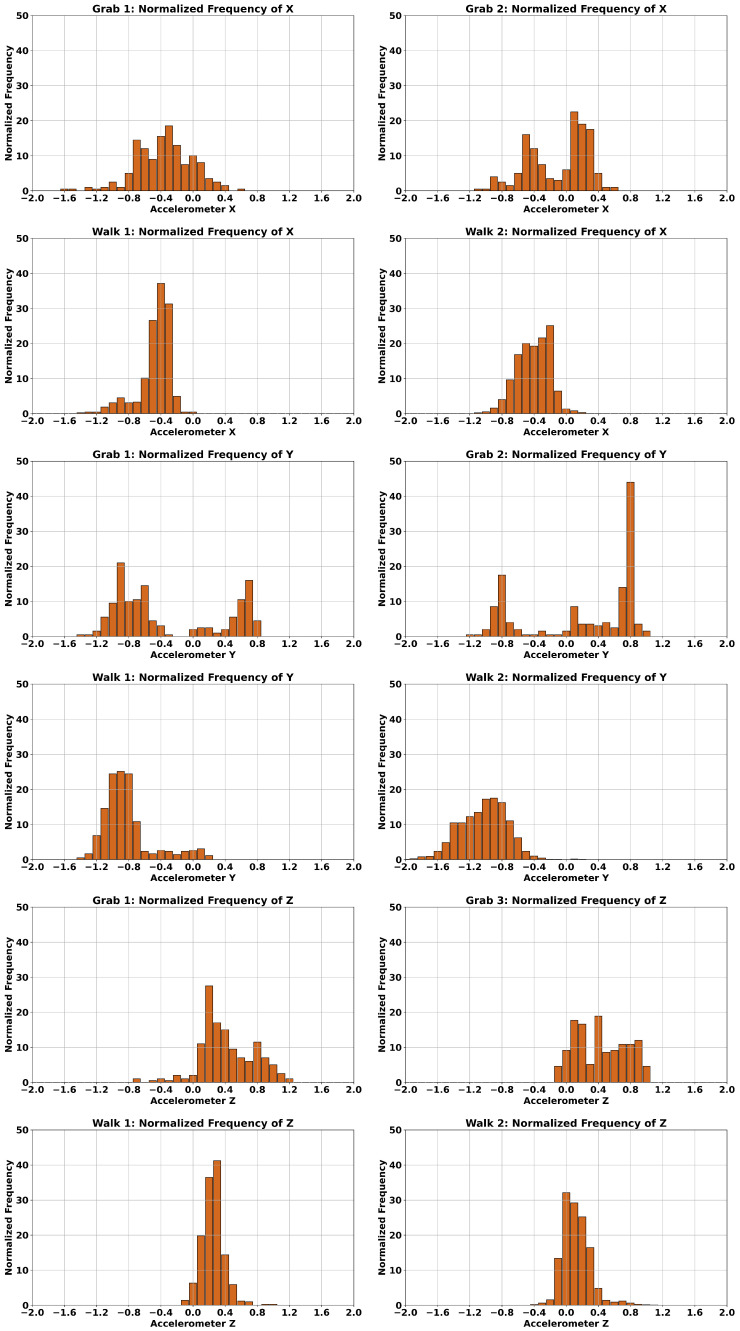
Frequency distributions of two instances of “Grabbing” and “Walking” across all axes of accelerometer data.

Figure 6 presents an analysis of the distribution patterns along the x-, y-, and z-axes of a single wrist-worn accelerometer for two activities: grabbing (grab 1 and grab 2) and walking (walk 1 and walk 2) of Figure 5. Prior to plotting, raw acceleration values were rounded to one decimal place. To ensure consistency across recordings with varying durations, all segments were normalized to a fixed 4-s window. The x- and y-axis ranges were also standardized across all subplots to enable direct visual comparison. In each plot, the horizontal axis represents binned acceleration values (ranging from −2 to 2), while the vertical axis denotes the normalized frequency (ranging from 0 to 50).

The first row in Figure 6 displays the x-axis acceleration distributions for two separate grabbing tasks. The second row shows corresponding distributions for two walking tasks along the x-axis. The third and fourth rows present distributions for the y-axis, while the fifth and sixth rows correspond to the z-axis.

Along the accelerometer x-axis (the first two rows), the grabbing tasks span between −1.2 to positive 0.7, approximately, while the two walking tasks have the tendency to be spanning between −1.2 to close to zero. Moreover, the walking task seems to have higher peaks (normalized frequency) compared to the two grabs.

More distinct patterns are observable in the two grabbing tasks (third row) and the two walking tasks (fourth row) along the accelerometer’s y-axis. The grabbing activities exhibit a bimodal distribution, with one peak on the negative side and another on the positive side of the horizontal axis. In contrast, the walking tasks tend to produce distributions that are skewed toward the negative side, indicating a consistent directional bias in arm movement during walking.

The accelerometer’s z-axis data also reveal notable differences between the grabbing activities (fifth row) and the walking tasks (sixth row). The distributions for the grabbing tasks are more dispersed, indicating greater variability in vertical motion, whereas the walking tasks exhibit sharper, narrower peaks with a more Gaussian-like shape, suggesting more consistent and repetitive movement patterns.

In addition, in Table 4, we present a summary for all the activities in Figure 5. It captures repeated instances of specific activities within the same session of Figure 5. For each instance segment of an activity, we computed mean, standard deviation, median, and zero-crossing rate (ZCR). ZCR measures the frequency at which the acceleration signal crosses the zero axis, providing insight into the intensity and variability of directional changes in motion. Notably, along the Z-axis, all the “Grabbing” tasks have a higher rate (0.125, 0.125, 0.201, and 0.143, respectively) of directional changes (ZCR) in acceleration, while no other activity has greater ZCR than 0.1. Also, in the accelerometer y-axis, the “Eating” task showed an elevated rate of ZCR (1.837), indicating more frequent movement variations than the other recorded tasks.

The distribution-based visualizations and the summary statistics of this section demonstrate that different activities produce distinctive acceleration patterns. This suggests a strong likelihood that accelerometer data can capture task-specific features. Consequently, the feasibility of distinguishing between functional activities solely from sensor data is reinforced, supporting our overarching objective of developing automated, sensor-based activity detection using sequence-to-sequence (Seq2Seq) learning models.

### 5.5. Additional Findings from the Data: Case Series Report

In addition to analyzing task-specific activities, accelerometer signals can also reveal broader trends in movement behavior over the course of a therapy session. In this section, we examine data from four patients, each exhibiting impairment on one hand, to investigate whether such impairment can be detected solely from accelerometer data. Our current results focus on the feasibility of using AI/ML techniques to automate the annotation of accelerometry data without an emphasis on characterizing the impairment features in patients with stroke.

Table 5 presents the demographic and clinical characteristics of the four patients who participated in the study. At the time of consent, patients p006 through p009 were aged 76, 68, 46, and 71 years, respectively. Patient p006 was a Hispanic African American female, while patients p007, p008, and p009 were Hispanic White males. All patients experienced ischemic strokes, with months post-stroke ranging from 7.5 (p007) to 126.9 (p006). In all cases, the dominant arm was affected, and impairment was concordant with hand dominance.

Motor function was evaluated using three standard clinical assessments: the NIH Stroke Scale (NIHSS) motor arm score, the Action Research Arm Test (ARAT), and the Upper Extremity Fugl-Meyer (UEFM) score. In the NIHSS motor arm subscale, higher scores indicate greater motor impairment (range: 0–4), with p006 showing the most severe impairment (score: 3) and p008 and p009 the least (score: 1). Conversely, ARAT (range: 0–57) and UEFM (range: 0–66) are positively oriented, higher scores indicate better motor function. Patient p006 had the lowest ARAT (9) and UEFM (22) scores on the impaired arm, reflecting significant functional limitations. In contrast, p008 and p009 exhibited relatively higher function with ARAT scores of 56 and 50, and UEFM scores of 60 and 58, respectively. All patients demonstrated full function on the unimpaired arm, with ARAT scores between 56 and 57 and UEFM scores of 66.

For each patient, we computed the standard deviation across all three axes of the accelerometer signals for both the impaired and non-impaired hands throughout the entire session. Standard deviation serves as a proxy for signal variability and movement intensity, offering insights into functional differences between the hands.

As shown in Table 6, patient p006, who presents with moderate impairment in one hand, exhibits the largest discrepancy in standard deviation between the impaired and non-impaired hands along the Z-axis. This observation aligns with the clinical assessments in Table 5, where patient p006 recorded the lowest ARAT and UEFM scores for the impaired arm, indicating significant functional limitations. The high asymmetry in movement intensity (difference = 0.3447) suggests reduced use of the impaired limb. The Z-axis, which captures vertical (up-down) motion, is typically more restricted in impaired limbs due to reduced range of motion and motor control. In contrast, patients p007 to p009, who had higher motor function scores, demonstrate much smaller differences in Z-axis standard deviation (ranging from 0.05 to 0.07), reflecting more symmetrical arm usage. This correspondence between clinical motor assessments and sensor-derived metrics reinforces the validity of accelerometry—particularly Z-axis data—as a quantitative indicator of motor asymmetry resulting from impairment.

## 6. Discussion

In this paper, we propose a novel AI-driven framework for generating natural language annotations from accelerometer data, as illustrated in Figure 1. The experiments presented focus on the feasibility of the framework’s key components. Our feasibility study demonstrates that it is possible to construct training samples for accelerometer data using video and sensor recordings collected in controlled laboratory environments. We also show that accelerometer signals contain sufficient variability and task-specific patterns to support the generation of meaningful annotations, indicating that natural language descriptions can be reliably mapped to time-series sensor data.

A major finding of this study is that characteristic movement signatures—such as dominant frequency components can be extracted from short segments of accelerometer data and used to distinguish between different types of activities. Furthermore, the analysis of standard deviation values revealed that impaired hands generally exhibited lower variability, particularly in the Z-axis, indicating reduced voluntary motion. These results suggest that such statistical features hold clinical relevance and could eventually serve as digital biomarkers for motor function assessment.

However, the approach is not without limitations. The success of the annotation pipeline heavily depends on the quality of the captions generated by the LLM and the reliability of the ML classifiers used to assign activity labels. Errors in either component can introduce label noise. Additionally, although our sensor setup was consistent, wearable sensor data is inherently sensitive to placement and orientation, which can affect generalization. The participant pool was also limited to a small number of stroke patients and healthy volunteers, meaning results may not yet generalize across broader populations or therapy types.

In terms of clinical potential, the framework demonstrates the feasibility of using low-cost wearables combined with automated annotation to support data-driven neurorehabilitation. This can reduce the burden on clinicians, allow for more frequent and objective assessments, and even support remote rehabilitation monitoring in home settings. Moreover, by linking specific movement patterns with therapy-relevant activities, the system could eventually serve as a quantitative decision-support tool for therapists.

Building on this pipeline, future work could involve deeper integration of LLMs and deep learning models with time-series data. Specifically, the idea of using a sequence-to-sequence (Seq2Seq) architecture to generate natural-language descriptions directly from accelerometer input appears technically feasible, assuming that a high-quality, labeled dataset is available. While challenges remain in curating such training data and tuning temporal models, the advancement of transformer-based architectures provides a strong foundation for this direction. With appropriate data alignment and augmentation strategies, it may become possible to generate human-readable descriptions of movement purely from sensor signals, bridging the gap between clinical observation and raw data streams.

## 7. Summary

This study presents an automated, multimodal framework for annotating and analyzing rehabilitation therapy sessions using synchronized video and accelerometer data. We addressed the challenge of limited labeled sensor data by leveraging LLMs to generate frame-level activity descriptions from video. These textual labels were then used to train machine learning classifiers that could accurately predict activity segments from video frames. Through frequency analysis, we demonstrated that distinct activities exhibit unique accelerometric signatures, making it feasible to distinguish functional movements based solely on sensor data.

## 8. Future Work

Building on the feasibility demonstrated in this study, our future work will focus on advancing a transformer-based sequence-to-sequence (Seq2Seq) architecture capable of generating natural language descriptions directly from accelerometer data. Our current results lay the groundwork by using manually aligned task descriptions generated via LLMs from video frames to label corresponding segments of sensor data. Our long-term goal is to train generative models that operate solely on sensor input. We plan to explore custom time-series encoders and adaptation strategies such as Low-Rank Adaptation (LoRA) to fine-tune existing language models for sensor-to-text generation.

Additionally, we aim to evaluate hierarchical text generation strategies that produce both fine-grained and high-level summaries of physical activity over varying time scales. Ongoing studies are enrolling participants with a wide range of stroke severities and duration since stroke to improve generalizability of the training data.

## Figures and Tables

**Table 1:** Some available open-source models for image-to-text generation.

Model	Primary Modality	Multimodal Support	Key Features
BLIP-2 [47]	Vision-Language	Yes	Pre-trained for vision-language bridging, strong captioning performance
LLaMA-3 [48]	Language	Indirect (via integration)	Can be paired with vision encoders for multimodal applications
LLaVA [49]	Language (with vision encoder)	Yes	Chat-style multimodal model with instruction-following capability
GIT [50]	Vision-Language	Yes	Unified model for image captioning and VQA using transformer
GPT-4V [51]	Language (closed weights)	Yes	Strong zero-shot capabilities on visual reasoning tasks
DUAL-LLM [52]	Vision-Language	Yes	Two-stream architecture optimized for image captioning
AGAIN [53]	Vision-Language	Yes	Focuses on grounding and alignment between vision and text

**Table 2:** LLaMA-3 generated descriptions, in three chunks (hand activity, background, and activity description), for frames relevant to different functional activities.

Frame	Hand Activity	Background	Activity Description
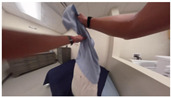	The subject’s hands are grasping a piece of fabric, possibly a shirt or dress, as they move through a room.	The background of the image is a white room with a bed and dresser, suggesting a bedroom or hospital room.	The subject appears to be in the process of putting on the garment, with their hands holding the fabric and their body moving through the room, possibly indicating that they are getting dressed or changing clothes.
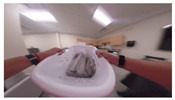	The hands are holding a laundry basket with a grey garment in it.	The hands are holding a laundry basket with a grey garment in it, in a room with white walls and a white ceiling.	The subject is in a home setting, doing a daily task of folding laundry, as they hold a laundry basket with a grey garment in it.
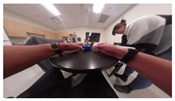	The first chunk describes the hand motion as “two hands reaching out towards a table from the bottom of the frame.”	The second chunk describes the background associated with the hands as “the hands are positioned in front of a round table in a room.”	The third chunk describes the subject’s activity in a home setting as "the subject is preparing a meal, likely in a kitchen, with the hands reaching for a bowl.”
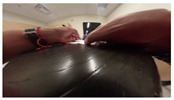	The first chunk describes the hand’s action: “The hand is holding a pen and writing on a piece of paper.”	The background shows a room with a white ceiling, a black TV, and a white wall.	The third chunk describes the activity in a home setting: “The subject is writing on a piece of paper, likely doing homework or taking notes in a home setting.”
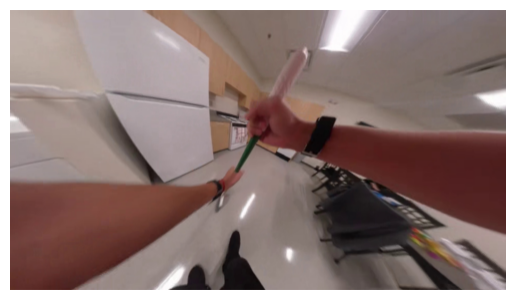	The subject is holding a green object in their hand, with their wrist bent in a way that suggests they are using it to point at something.	The background appears to be a room with white walls and a white ceiling, possibly a classroom or office setting.	The subject is holding a green object in their hand, with their wrist bent in a way that suggests they are using it to point at something.....

**Table 3:** GPT-4o responses to two different prompts for images associated with various daily activities. The model accurately identifies instances of grasping or holding, highlighting its potential for detailed functional analysis.

Frame	Prompt 1	Prompt 2
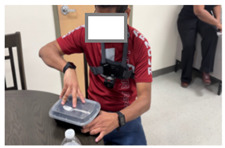	The person in the image appears to be engaged in household activities, specifically opening a plastic container at a table.	Left hand: Grabbing. What is the left hand holding: A container lid. Right hand: Grabbing. What is the right hand holding: A container
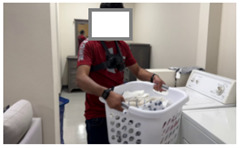	The person is doing laundry. They are carrying a laundry basket filled with clothes.	Left hand: Grabbing. What is the left hand holding: A laundry basket. Right hand: Grabbing. What is the right hand holding: A laundry basket.
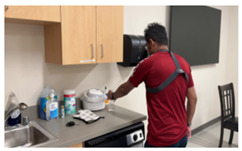	The person in the image appears to be engaged in a kitchen-related activity. They are standing at a counter, placing or handling a bottle. This setup, with cleaning supplies and dishware present, suggests they might be tidying up or preparing the area for another task. The presence of a camera harness indicates that their actions are being recorded, likely for documenting or broadcasting their activities.	Left hand: Grabbing. What is the left hand holding: A transparent bottle. Right hand: Not grabbing. What is the right hand holding: Nothing.

**Table 4:** Summary of statistical features from accelerometer signals, grouped by axis and categorized by task. Distinct movement patterns are evident from Z-axis ZCR analysis, with the “Grabbing” task showing higher ZCR values than other tasks along the Z-axis.

Task	Axis X				Axis Y				Axis Z			
	**Mean**	**std**	**Median**	**ZCR**	**Mean**	**std**	**Median**	**ZCR**	**Mean**	**std**	**Median**	**ZCR**
Grab 1	−0.358	0.344	−0.346	2.125	−0.311	0.685	−0.626	0.250	0.418	0.333	0.358	0.125
Grab 2	−0.096	0.376	0.069	2.500	0.209	0.690	0.627	0.375	0.550	0.297	0.526	0.125
Grab 3	−0.274	0.139	−0.276	0.805	−0.983	0.181	−0.953	0.000	0.128	0.163	0.119	0.201
Grab 4	−0.375	0.190	−0.399	0.286	−0.634	0.428	−0.787	0.857	0.427	0.314	0.393	0.143
Laundry	−0.562	0.376	−0.580	1.394	−0.435	0.433	−0.448	0.758	0.409	0.332	0.354	0.015
Eat	−0.454	0.515	−0.566	1.160	0.144	0.415	0.145	1.837	0.553	0.277	0.530	0.032
Walk 1	−0.470	0.206	−0.425	0.000	−0.823	0.311	−0.887	0.529	0.244	0.139	0.249	0.059
Walk 2	−0.408	0.207	−0.396	0.284	−1.027	0.287	−0.999	0.023	0.120	0.173	0.106	0.011
Walk 3	−0.376	0.188	−0.369	0.800	−0.998	0.253	−0.965	0.000	−0.009	0.156	−0.027	0.015

**Table 5:** Summary of demographic and clinical profiles of patients enrolled in the study. Race AA refers to African American. Concordant stroke indicates that the dominant upper extremity (determined using the Edinburgh Handedness Inventory) is more impaired. ARAT refers to Action Research Arm Test. UEFM is Upper-Extremity Fugl-Meyer. PID is the Participant ID.

PID	p006	p007	p008	p009
Age (years)	76	68	46	71
Sex	Female	Male	Male	Male
Race	AA	White	White	White
Ethnicity	Hispanic	Hispanic	Hispanic	Hispanic
Stroke type	Ischemic	Ischemic	Ischemic	Ischemic
Months post-stroke	126.9	7.5	26.2	37.5
Affected arm	Dominant	Dominant	Dominant	Dominant
Concordance	Concordant	Concordant	Concordant	Concordant
NIHSS motor arm (Impaired)	3	2	1	1
NIHSS motor arm (Unimpaired)	0	0	0	0
ARAT (Impaired)	9	40	56	50
ARAT (Unimpaired)	56	57	57	57
UEFM (Impaired)	22	48	60	58
UEFM (Unimpaired)	66	66	66	66

**Table 6:** Standard deviation of Z-axis accelerometer signals for the impaired and non-impaired hands across all participants. The ”Difference” column represents the absolute difference in standard deviation between the two hands, highlighting the asymmetry in movement intensity.

Patient ID	Std Dev (Working Hand)	Std Dev (Impaired Hand)	Difference
p006	0.754	0.409	0.344
p007	0.498	0.428	0.070
p008	0.502	0.449	0.053
p009	0.478	0.427	0.050

## Data Availability

The raw data supporting the conclusion of this article will be made available by the authors upon request.

## References

[1] Kleindorfer D.O., Towfighi A., Chaturvedi S., Cockroft K.M., Gutierrez J., Lombardi-Hill D., Kamel H., Kernan W.N., Kittner S.J., Leira E.C. (2021). 2021 Guideline for the Prevention of Stroke in Patients with Stroke and Transient Ischemic Attack: A Guideline from the American Heart Association/American Stroke Association. Stroke.

[2] Virani S.S., Alonso A., Benjamin E.J., Bittencourt M.S., Callaway C.W., Carson A.P., Chamberlain A.M., Chang A.R., Cheng S., Delling F.N. (2020). Heart Disease and Stroke Statistics-2020 Update: A Report from the American Heart Association. Circulation.

[3] Geed S., Feit P., Edwards D.F., Dromerick A.W. (2021). Why Are Stroke Rehabilitation Trial Recruitment Rates in Single Digits?. Front. Neurol..

[4] Simpson L.A., Hayward K.S., McPeake M., Field T.S., Eng J.J. (2021). Challenges of Estimating Accurate Prevalence of Arm Weakness Early After Stroke. Neurorehabil. Neural Repair.

[5] Young B.M., Holman E.A., Cramer S.C. (2023). Rehabilitation Therapy Doses Are Low After Stroke and Predicted by Clinical Factors. Stroke.

[6] Barth J., Geed S., Mitchell A., Brady K.P., Giannetti M.L., Dromerick A.W., Edwards D.F. (2023). The Critical Period After Stroke Study (CPASS) Upper Extremity Treatment Protocol. Arch. Rehabil. Res. Clin. Transl..

[7] Kwakkel G., Stinear C., Essers B., Munoz-Novoa M., Branscheidt M., Cabanas-Valdés R., Lakičević S., Lampropoulou S., Luft A.R., Marque P. (2023). Motor rehabilitation after stroke: European Stroke Organisation (ESO) consensus-based definition and guiding framework. Eur. Stroke J..

[8] Bernhardt J., Hayward K.S., Kwakkel G., Ward N.S., Wolf S.L., Borschmann K., Krakauer J.W., Boyd L.A., Carmichael S.T., Corbett D. (2017). Agreed definitions and a shared vision for new standards in stroke recovery research: The Stroke Recovery and Rehabilitation Roundtable taskforce. Int. J. Stroke.

[9] Wolf S.L., Kwakkel G., Bayley M., McDonnell M.N. (2016). Best practice for arm recovery post stroke: An international application. Physiotherapy.

[10] Steins D., Dawes H., Esser P., Collett J. (2014). Wearable accelerometry-based technology capable of assessing functional activities in neurological populations in community settings: A systematic review. J. Neuroeng. Rehabil..

[11] Jim E.A., Utsha M.A.H., Aurna F.N., Choudhury A., Hoque M.A. Towards Safer Aging: A Comprehensive Edge Computing Approach to Unconsciousness and Fall Detection. Proceedings of the 2025 International Conference on Electrical, Computer and Communication Engineering (ECCE).

[12] Geed S. (2024). Towards measuring the desired neurorehabilitation outcomes directly with accelerometers and machine learning. Dev. Med. Child Neurol..

[13] Geed S., Grainger M.L., Mitchell A., Anderson C.C., Schmaulfuss H.L., Culp S.A., McCormick E.R., McGarry M.R., Delgado M.N., Noccioli A.D. (2023). Concurrent validity of machine learning-classified functional upper extremity use from accelerometry in chronic stroke. Front. Physiol..

[14] Sequeira S.B., Grainger M.L., Mitchell A.M., Anderson C.C., Geed S., Lum P., Giladi A.M. (2022). Machine Learning Improves Functional Upper Extremity Use Capture in Distal Radius Fracture Patients. Plast. Reconstr. Surgery Global Open.

[15] Barth J., Geed S., Mitchell A., Lum P.S., Edwards D.F., Dromerick A.W. (2020). Characterizing upper extremity motor behavior in the first week after stroke. PLoS ONE.

[16] Mathew S.P., Dawe J., Musselman K.E., Petrevska M., Zariffa J., Andrysek J., Biddiss E. (2024). Measuring functional hand use in children with unilateral cerebral palsy using accelerometry and machine learning. Dev. Med. Child Neurol..

[17] Tran T., Chang L.C., Lum P. Functional Arm Movement Classification in Stroke Survivors Using Deep Learning with Accelerometry Data. Proceedings of the IEEE International Conference on Big Data.

[18] Dobkin B.H., Martinez C. (2018). Wearable Sensors to Monitor, Enable Feedback, and Measure Outcomes of Activity and Practice. Curr. Neurol. Neurosci. Rep..

[19] Aziz O., Park E.J., Mori G., Robinovitch S.N. (2014). Distinguishing the causes of falls in humans using an array of wearable tri-axial accelerometers. Gait Posture.

[20] Gebruers N., Vanroy C., Truijen S., Engelborghs S., De Deyn P.P. (2010). Monitoring of physical activity after stroke: A systematic review of accelerometry-based measures. Arch. Phys. Med. Rehabil..

[21] Clark E., Podschun L., Church K., Fleagle A., Hull P., Ohree S., Springfield M., Wood S. (2023). Use of accelerometers in determining risk of falls in individuals post-stroke: A systematic review. Clin. Rehabil..

[22] Peters D.M., O’Brien E.S., Kamrud K.E., Roberts S.M., Rooney T.A., Thibodeau K.P., Balakrishnan S., Gell N., Mohapatra S. (2021). Utilization of wearable technology to assess gait and mobility post-stroke: A systematic review. J. NeuroEngineering Rehabil..

[23] Nieto E.M., Lujan E., Mendoza C.A., Arriaga Y., Fierro C., Tran T., Chang L.-C., Gurovich A.N., Lum P.S., Geed S. (2025). Accelerometry and the Capacity–Performance Gap: Case Series Report in Upper-Extremity Motor Impairment Assessment Post-Stroke. Bioengineering.

[24] Urbin M.A., Waddell K.J., Lang C.E. (2015). Acceleration metrics are responsive to change in upper extremity function of stroke survivors. Arch. Phys. Med. Rehabil..

[25] Lum P.S., Shu L., Bochniewicz E.M., Tran T., Chang L.C., Barth J., Dromerick A.W. (2020). Improving Accelerometry-Based Measurement of Functional Use of the Upper Extremity After Stroke: Machine Learning Versus Counts Threshold Method. Neurorehabilit. Neural Repair.

[26] Srinivasan S., Amonkar N., Kumavor P.D., Bubela D. (2024). Measuring Upper Extremity Activity of Children with Unilateral Cerebral Palsy Using Wrist-Worn Accelerometers: A Pilot Study. Am. J. Occup. Ther..

[27] Dawe J., Yang J.F., Fehlings D., Likitlersuang J., Rumney P., Zariffa J., Musselman K.E. (2019). Validating Accelerometry as a Measure of Arm Movement for Children with Hemiplegic Cerebral Palsy. Phys. Ther..

[28] Bailey D.P., Ahmed I., Cooper D.L., Finlay K.A., Froome H.M., Nightingale T.E., Romer L.M., Goosey-Tolfrey V.L., Ferrandino L. (2025). Validity of a wrist-worn consumer-grade wearable for estimating energy expenditure, sedentary behaviour, and physical activity in manual wheelchair users with spinal cord injury. Disabil. Rehabil. Assist. Technol..

[29] Khan M.U.G., Zhang L., Gotoh Y. Human Focused Video Description. Proceedings of the IEEE International Conference on Computer Vision Workshops (ICCV Workshops).

[30] Hanckmann P., Schutte K., Burghouts G.J. Automated Textual Descriptions for a Wide Range of Video Events with 48 Human Actions. Proceedings of the Computer Vision—ECCV 2012. Workshops and Demonstrations.

[31] Ramanishka V., Das A., Park D.H., Venugopalan S., Hendricks L.A., Rohrbach M., Saenko K. Multimodal Video Description. Proceedings of the 24th ACM International Conference on Multimedia (MM ’16).

[32] Hori C., Hori T., Lee T.-Y., Zhang Z., Harsham B., Hershey J.R., Marks T.K., Sumi K. Attention-Based Multimodal Fusion for Video Description. Proceedings of the IEEE International Conference on Computer Vision.

[33] Thomason J., Venugopalan S., Guadarrama S., Saenko K., Mooney R. Integrating Language and Vision to Generate Natural Language Descriptions of Videos in the Wild. Proceedings of the COLING 2014: The 25th International Conference on Computational Linguistics: Technical Papers.

[34] Wang X., Chen W., Wu J., Wang Y.-F., Wang W.Y. Video Captioning via Hierarchical Reinforcement Learning. Proceedings of the IEEE Conference on Computer Vision and Pattern Recognition, Salt Lake City.

[35] Li L., Hu K. Text Description Generation from Videos via Deep Semantic Models. Proceedings of the 2021 Asia-Pacific Signal and Information Processing Association Annual Summit and Conference (APSIPA ASC).

[36] Chen L., Wei X., Li J., Dong X., Zhang P., Zang Y., Chen Z., Duan H., Tang Z., Yuan L. (2024). ShareGPT4Video: Improving Video Understanding and Generation with Better Captions. Advances in Neural Information Processing Systems.

[37] Allappa S.S., Thenkanidiyoor V., Dinesh D.A. Video Activity Recognition Using Sequence Kernel Based Support Vector Machines. Proceedings of the International Conference on Pattern Recognition Applications and Methods.

[38] de Almeida Maia H., Ttito Concha D., Pedrini H., Tacon H., de Souza Brito A., de Lima Chaves H., Bernardes Vieira M., Moraes Villela S. (2020). Action Recognition in Videos Using Multi-Stream Convolutional Neural Networks. Deep Learning Applications.

[39] Kulbacki M., Segen J., Chaczko Z., Rozenblit J.W., Kulbacki M., Klempous R., Wojciechowski K. (2023). Intelligent Video Analytics for Human Action Recognition: The State of Knowledge. Sensors.

[40] Ullah H.A., Letchmunan S., Zia M.S., Butt U.M., Hassan F.H. (2021). Analysis of Deep Neural Networks for Human Activity Recognition in Videos—A Systematic Literature Review. IEEE Access.

[41] Soleimani F., Khodabandelou G., Chibani A., Amirat Y. (2025). Activity Recognition via Multimodal Large Language Models and Riemannian Optimization. HAL Preprint. https://hal.science/hal-04943796v1/file/Main_V0.pdf.

[42] Lara O.D., Labrador M.A. (2012). A Survey on Human Activity Recognition Using Wearable Sensors. IEEE Commun. Surv. Tutor..

[43] Shakya S.R., Zhang C., Zhou Z. (2018). Comparative Study of Machine Learning and Deep Learning Architecture for Human Activity Recognition Using Accelerometer Data. Int. J. Mach. Learn. Comput..

[44] Chernbumroong S., Atkins A.S., Yu H. Activity Classification Using a Single Wrist-Worn Accelerometer. Proceedings of the 2011 5th International Conference on Software, Knowledge Information, Industrial Management and Applications (SKIMA) Proceedings.

[45] Panwar M., Dyuthi S.R., Prakash K.C., Biswas D., Acharyya A., Maharatna K., Gautam A., Naik G.R. CNN Based Approach for Activity Recognition Using a Wrist-Worn Accelerometer. Proceedings of the 2017 39th Annual International Conference of the IEEE Engineering in Medicine and Biology Society (EMBC).

[46] Uswatte G., Taub E., Morris D.P.P.T., Light K.P.P.T., Thompson P.A. (2006). The Motor Activity Log-28: Assessing Daily Use of the Hemiparetic Arm after Stroke. Neurology.

[47] Li J., Li D., Savarese S., Hoi S. BLIP-2: Bootstrapping Language-Image Pre-Training with Frozen Image Encoders and Large Language Models. Proceedings of the International Conference on Machine Learning.

[48] Grattafiori A., Dubey A., Jauhri A., Pandey A., Kadian A., Al-Dahle A., Letman A., Mathur A., Schelten A., Vaughan A. (2024). The LLaMA 3 Herd of Models. arXiv.

[49] Jin Y., Li J., Zhang J., Hu J., Gan Z., Tan X., Liu Y., Wang Y., Wang C., Ma L. LLaVA-VSD: Large Language-and-Vision Assistant for Visual Spatial Description. Proceedings of the 32nd ACM International Conference on Multimedia.

[50] Wang J., Yang Z., Hu X., Li L., Lin K., Gan Z., Liu Z., Liu C., Wang L. (2022). GIT: A Generative Image-to-Text Transformer for Vision and Language. arXiv.

[51] Ding N., Tang Y., Fu Z., Xu C., Han K., Wang Y. (2023). GPT4IMAGE: Can Large Pre-Trained Models Help Vision Models on Perception Tasks?. arXiv.

[52] Xia M., Wu Z. Dual-Encoder-Based Image-Text Fusion Algorithm. Proceedings of the International Conference on Image Processing and Artificial Intelligence (ICIPAl 2024).

[53] Tran M.-N., To T.-A., Thai V.-N., Cao T.-D., Nguyen T.-T. AGAIN: A Multimodal Human-Centric Event Retrieval System Using Dual Image-to-Text Representations. Proceedings of the 12th International Symposium on Information and Communication Technology.

[54] Hu E., Shen Y., Wallis P., Allen-Zhu Z., Li Y., Wang S., Wang L., Chen W. (2021). LoRA: Low-Rank Adaptation of Large Language Models. arXiv.

[55] Zhou H., Zhang S., Peng J., Zhang S., Li J., Xiong H., Zhang W. Informer: Beyond Efficient Transformer for Long Sequence Time-Series Forecasting. Proceedings of the AAAI Conference on Artificial Intelligence.

